# Supplementary material for: Pupillary responses to affective words in bilinguals’ first versus second language
Source: PLoS One. 2019 Apr 23;14(4):e0210450. doi: 10.1371/journal.pone.0210450 (PMC6478288; doi:10.1371/journal.pone.0210450)
Supplement: S1 Appendix — The selected 30 High- and 30 Low Arousal words per language, in alphabetical order. (DOCX) [file pone.0210450.s001.docx]

**Appendix S1: Selected Stimuli**

The selected 30 High- and 30 Low Arousal words per language, in alphabetical order.

| **English** | | **Finnish** | | **German** | |
| --- | --- | --- | --- | --- | --- |
| **High Arousal** | **Low Arousal** | **High Arousal** | **Low Arousal** | **High Arousal** | **Low Arousal** |
| ADVENTURE | ALLEY | AHDINKO | ASUKAS | ARSCH | ANSATZ |
| ARSONIST | APPLIANCE | AHDISTUS | HYYTELÖ | ELEND | BECHER |
| ATTACK | ASLEEP | ÄITI | KAMARI | ERDBEBEN | BESTECK |
| BOMBING | BARREL | HÄIRINTÄ | KANGAS | FURCHT | DECKEL |
| BULLSHIT | BREAD | HERJAUS | KATU | GENIE | ERBSE |
| CHAOS | CABINET | HURMIO | KÄYTÄVÄ | GEWALT | HEFTER |
| CROCODILE | CARDBOARD | IHASTUS | KOKO | GIFT | INHALT |
| DEATH | COLUMN | ILO | KUJA | HASS | KANNE |
| DIE | CONTENT | INTOHIMO | LAITE | HENKER | KARTE |
| EARTHQUAKE | CORNER | KÄRSIMYS | LASI | HÖHEPUNKT | KARTON |
| EROTIC | CUP | KASVAIN | LEIPÄ | HURE | KLAMMER |
| EXCITING | DOOR | KIDUTTAJA | MAITO | INTENSIV | LEITER |
| FOREPLAY | DULL | KOSTO | MOOTTORI | LAWINE | LÖFFEL |
| HATE | FABRIC | LAHJONTA | NURKKA | LEICHE | QUADRAT |
| INTENSITY | FLAG | MYRKYTYS | OSA | LEICHNAM | REGAL |
| LIGHTNING | HONEY | ORJUUS | PATSAS | LÜGE | SATZ |
| LOVER | JELLY | PAHUUS | PÖYTÄ | MASSAKER | SCHALTER |
| PASSION | LAWN | PAINOSTUS | PYLVÄS | MORD | SEQUENZ |
| PLAGUE | LEVEL | PELKO | REITTI | MÖRDER | SIEB |
| RAGE | MILK | PERHE | SIENI | NAZI | SOHLE |
| RIOT | PEA | RAISKAUS | SISÄLTÖ | PANIK | SORTE |
| RISK | SLIME | ROMANSSI | SOLU | RACHE | STAPEL |
| SCREAM | SQUARE | RÖYHKEYS | SUIHKE | SCHLACHT | STOFF |
| SEDUCTION | STATUE | SEKSI | TAPA | SCHLAMPE | STRAßE |
| SEX | SWAMP | SUUDELMA | TIETOKONE | STERBEN | STUFE |
| SEXUAL | SYLLABLE | SUUTTUMUS | TUOLI | TRENNUNG | STUHL |
| SNAKE | TABLE | TAPPO | TYNNYRI | TUMULT | TISCH |
| TERRORISM | TEA | ULVONTA | VAIHE | UNRECHT | WIESE |
| TORNADO | VEHICLE | VALITTAJA | VESI | VERHAFTEN | WINKEL |
| VENOMOUS | WATER | VIHA | YDIN | WOLLUST | ZEILE |
