# Supplementary material for: Pupillary responses to affective words in bilinguals’ first versus second language
Source: PLoS One. 2019 Apr 23;14(4):e0210450. doi: 10.1371/journal.pone.0210450 (PMC6478288; doi:10.1371/journal.pone.0210450)
Supplement: S2 Appendix — Information about the Principal Component Analysis on item characteristics. (DOCX) [file pone.0210450.s002.docx]

**Appendix S2, Principal Components of Control Variables**

Scores for length (in characters and syllables), valence, frequency, and abstractness per word (N=180 across languages and arousal categories) were entered into a Principal Component Analysis (PCA) to condense them into a smaller set of orthogonal factors, based on the *correlation matrix* of the original variables (standardized scaling). As extraction criterion, we aimed for the smallest number of principal components necessary to preserve at least 80% of the variability in the original control variables. This resulted in three principal components which together explained 84% of the original variance. Table S2A shows communalities (what proportion of variance in each of the original variables is captured by the three extracted factors) and Table S2B the factor loadings (correlations between the original variables and each of the three extracted factors) in Equamax-rotated space.

**Table S2A.** Communalities Resulting from 3-Factor Solution

| **Original Variable** | **% Variance Captured** |
| --- | --- |
| Letters | 81 |
| Syllables | 82 |
| Valence | 92 |
| Abstractness | 92 |
| Frequency | 74 |

**Table S2B.** Factor Loadings after Equamax Rotation

|  | Principal Component | | |
| --- | --- | --- | --- |
|  | PC1 | PC2 | PC3 |
| Letters | **.89** | −.08 | .12 |
| Syllables | **.89** | .04 | .16 |
| Valence | −.01 | **.95** | −.16 |
| Abstractness | .09 | −.13 | **.94** |
| Frequency | **−.75** | .29 | .28 |

Absolute factor loadings greater than .7 are highlighted in bold in Table S2B. As becomes evident, both Letters and Syllables loaded strongly positively, and Frequency strongly negatively on the first principal component (henceforth labelled *PC1:LenFreq*; more positive scores on this component index longer, less frequent words). Valence loaded strongly positively on the second, and Abstractness strongly positively on the third principal component (henceforth labelled *PC2:Valence* and *PC3:Abstractness*, respectively).
