# Supplementary material for: Pupillary responses to affective words in bilinguals’ first versus second language
Source: PLoS One. 2019 Apr 23;14(4):e0210450. doi: 10.1371/journal.pone.0210450 (PMC6478288; doi:10.1371/journal.pone.0210450)
Supplement: S3 Appendix — Supplemental information on random effects in the area-under-curve analyses. (DOCX) [file pone.0210450.s003.docx]

**Inferential Analysis, Supplemental Information on Random Effects**

**Table S3A.** Random effect variance estimates for the area-under-the-curve analysis considering **bilingual participants only**. The notation “x | y” indicates that effect x is nested within variable y. Estimates of zero suggest redundant parameters or insufficient data for their estimation, respectively. L1 = first language of participant; TL = Target Language; WT = Word Type; PC1 = principal component with strong length and frequency loadings (*PC1:LenFreq*); PC2 = principal component with strong valence loadings (*PC2:Valence*); PC3 = principal component with strong abstractness loadings (*PC3:Abstractness*).

| Random Effect | | Estimate | SE | Wald Z | *p* |
| --- | --- | --- | --- | --- | --- |
|  |  |  |  |  |  |
| Residual | | 14552.6 | 239.6 | 60.743 | .000 |
| Subj \| L1 |  | 547.3 | 170.4 | 3.212 | .001 |
| Subj × TL \| L1 |  | 435.0 | 121.8 | 3.571 | .000 |
| Subj × WT \| L1 |  | 0.0 | 0.0 | . | . |
| Subj × TL × WT \| L1 |  | 0.0 | 0.0 | . | . |
| Item \| TL |  | 196.4 | 57.3 | 3.427 | .001 |
| PC1 |  | 0.0 | 0.0 | . | . |
| PC2 |  | 0.0 | 0.0 | . | . |
| PC3 |  | 18.1 | 32.2 | .561 | .575 |
| TL × PC1 |  | 13.0 | 20.4 | .637 | .524 |
| TL × PC2 |  | 44.1 | 51.8 | .851 | .395 |
| TL × PC3 |  | 0.0 | 0.0 | . | . |

**Notes:**

1. Since sampling distributions of variance estimates tend to be strongly asymmetric, the above *SE*s (and corresponding significance tests) must be treated with caution.
2. Items were selected such that variability in the control predictors (letters, syllables, frequency, valence, and abstractness) was kept fairly moderate overall. This might explain why the principal components (*PC1*, *PC2*, *PC3*) contributed relatively small variance components to the model fits.

**Table S3B.** Random effect variance estimates for the area-under-the-curve analysis considering **English as test language only**. The notation “x | y” indicates that effect x is nested within variable y. Estimates of zero suggest redundant parameters (or insufficient data for their estimation, respectively). L1 = first language of participant; TL = Target Language; WT = Word Type; PC1 = principal component with strong length and frequency loadings (*PC1:LenFreq*); PC2 = principal component with strong valence loadings (*PC2:Valence*); PC3 = principal component with strong abstractness loadings (*PC3:Abstractness*).

| Random Effect | | Estimate | SE | Wald Z | *p* |
| --- | --- | --- | --- | --- | --- |
|  |  |  |  |  |  |
| Residual | | 16065.7 | 303.8 | 52.887 | .000 |
| Subj \| L1 |  | 1208.7 | 216.6 | 5.580 | .000 |
| Subj × WT \| L1 |  | 0.0 | 0.0 | . | . |
| Item |  | 192.9 | 68.5 | 2.818 | .005 |
| PC1 |  | 17.0 | 31.7 | .535 | .593 |
| PC2 |  | 33.4 | 85.2 | .392 | .695 |
| PC3 |  | 0.3 | 12.9 | .021 | .984 |
| L1 × PC1 |  | 0.0 | 0.0 | . | . |
| L1 × PC2 |  | 50.7 | 58.9 | .861 | .389 |
| L1 × PC3 |  | 0.0 | 0.0 | . | . |

**Notes:**

1. Since sampling distributions of variance estimates tend to be strongly asymmetric, the above *SE*s (and corresponding significance tests) must be treated with caution.
2. Items were selected such that variability in the control predictors (letters, syllables, frequency, valence, and abstractness) was kept fairly moderate overall. This might explain why the principal components (*PC1*, *PC2*, *PC3*) contributed relatively small variance components to the model fits.
